# AI-assisted compressed sensing and parallel imaging sequences for MRI of patients with nasopharyngeal carcinoma: comparison of their capabilities in terms of examination time and image quality

**DOI:** 10.1007/s00330-023-09742-6

**Published:** 2023-05-23

**Authors:** Haibin Liu, Dele Deng, Weilong Zeng, Yingyi Huang, Chunling Zheng, Xinyang Li, Hui Li, Chuanmiao Xie, Haoqiang He, Guixiao Xu

**Affiliations:** 1 Department of Radiology, State Key Laboratory of Oncology in South China, Guangdong Key Laboratory of Nasopharyngeal Carcinoma Diagnosis and Therapy, Sun Yat-sen University Cancer Center, Guangzhou, 510060 People’s Republic of China; 2 United Imaging Healthcare, Shanghai, People’s Republic of China

**Keywords:** Compressed sensing, Parallel imaging, Magnetic resonance imaging, Nasopharyngeal carcinoma

## Abstract

**Objective:**

To compare examination time and image quality between artificial intelligence (AI)–assisted compressed sensing (ACS) technique and parallel imaging (PI) technique in MRI of patients with nasopharyngeal carcinoma (NPC).

**Methods:**

Sixty-six patients with pathologically confirmed NPC underwent nasopharynx and neck examination using a 3.0-T MRI system. Transverse T2-weighted fast spin-echo (FSE) sequence, transverse T1-weighted FSE sequence, post-contrast transverse T1-weighted FSE sequence, and post-contrast coronal T1-weighted FSE were obtained by both ACS and PI techniques, respectively. The signal-to-noise ratio (SNR), contrast-to-noise ratio (CNR), and duration of scanning of both sets of images analyzed by ACS and PI techniques were compared. The images from the ACS and PI techniques were scored for lesion detection, margin sharpness of lesions, artifacts, and overall image quality using the 5-point Likert scale.

**Results:**

The examination time with ACS technique was significantly shorter than that with PI technique (*p* < 0.0001). The comparison of SNR and CNR showed that ACS technique was significantly superior with PI technique (*p* < 0.005). Qualitative image analysis showed that the scores of lesion detection, margin sharpness of lesions, artifacts, and overall image quality were higher in the ACS sequences than those in the PI sequences (*p* < 0.0001). Inter-observer agreement was evaluated for all qualitative indicators for each method, in which the results showed satisfactory-to-excellent agreement (*p* < 0.0001).

**Conclusion:**

Compared with the PI technique, the ACS technique for MR examination of NPC can not only shorten scanning time but also improve image quality.

**Clinical relevance statement:**

The artificial intelligence (AI)–assisted compressed sensing (ACS) technique shortens examination time for patients with nasopharyngeal carcinoma, while improving the image quality and examination success rate, which will benefit more patients.

**Key Points:**

• *Compared with the parallel imaging (PI) technique, the artificial intelligence (AI)–assisted compressed sensing (ACS) technique not only reduced examination time, but also improved image quality*.

• *Artificial intelligence (AI)–assisted compressed sensing (ACS) pulls the state-of-the-art deep learning technique into the reconstruction procedure and helps find an optimal balance of imaging speed and image quality*.

## Introduction

Nasopharyngeal carcinoma (NPC) is an endemic disease in Southeast Asia, especially in some southern provinces of mainland China [1]. Magnetic resonance imaging (MRI) is widely used in the diagnosis, staging, and efficacy evaluation of NPC. Compared with computed tomography (CT), MRI can better identify early-stage NPC (stages I–II) and has superior sensitivity and specificity for discriminating adjacent soft tissue invasion, skull base invasion, cranial nerve invasion, and retropharyngeal lymph node involvement [2–4].

Although the advantages of MRI are noticeable, the time-consuming feature of MRI can lead to patients’ fatigue and motion artifacts, reducing the quality of images. K-space under-sampling is currently the main method for reducing MRI scan time [5]. There are three main approaches to perform k-space under-sampling, including Half Fourier (HF) imaging, parallel imaging (PI), and compression sensing (CS) [6]. The HF technique is based on the Hermitian conjugate symmetry of the k-space. Only half of the k-space data are acquired in the phase encoding direction under ideal conditions, and the other half can be calculated and filled according to the conjugate symmetry of the k-space [7–9]. The deficiency of HF is that Gibbs artifacts are generated and signal-to-noise ratio (SNR) of an image decreases inevitably. The PI technique relies on the use of a receiver coil array to collect under-sampled k-space data and on specialized algorithms to reconstruct the complete field-of-view (FOV) images [10, 11]. PI is a commonly used acceleration tool in clinical applications, while image quality at high-acceleration factors may be reduced by noise amplification and under-sampled artifacts. The CS technique provides a new approach to recover imaging data from under-sampled k-space through the exploitation of sparsity. A small number of signals acquired by incoherence sampling are reconstructed with a high-probability using a reconstruction algorithm, and finally higher quality MR images are obtained by the Fourier transform [12–14].

In recent years, CS has been widely used as a new under-sampled k-space method. However, its insufficient sparsity may lead to noise-like aliasing artifacts when excessive acceleration factors are employed. Therefore, some MR vendors have introduced deep learning reconstruction (DLR) to improve image quality [15–17], such as the Advanced intelligent Clear IQ Engine (AiCE) developed by Canon Medical Systems Corporation [18]. A new acceleration method, namely artificial intelligence (AI)–assisted compressed sensing (ACS), was developed by United Imaging Intelligence (UII) and United Imaging Healthcare (UIH), incorporating CS, HF, and PI to innovatively introduce deep learning neural networks as AI modules into the reconstruction process [19].

MRI is the most appropriate method for localization and qualitative and staging diagnosis of NPC [20–23]. T2-weighted fast spin-echo (FSE), T1-weighted FSE, and contrast-enhanced T1-weighted FSE are key sequences in MRI of NPC [24]. Conventional MRI scans of NPC are dominated by FSE sequences with PI [25], and in some sequences, a combination of fat suppression is required to determine whether the tumor has bone marrow infiltration of the skull base [26, 27]. In addition, the MRI of NPC must be scanned in combination with the nasopharynx and neck to facilitate clinical staging. The longer total time of MRI scans due to the combined nasopharyngeal and neck scan and the adoption of FSE sequences with a longer time, resulting in failure of some patients in completion of the examination because of intolerance. The present study aimed to apply the innovative ACS technique to FSE sequences to explore the capabilities of ACS in improving the time and quality of MRI of NPC.

## Methods

### Study population

The study was approved by the institutional review board of our hospital (Approval No. B2020-417). From August 2021 to December 2021, a total of 72 patients with confirmed or suspected NPC underwent nasopharynx and neck examination using a 3.0-T MR system. The inclusion criteria were as follows: (1) newly diagnosed, untreated patients with NPC; (2) patients who aged  ≥ 18 years old. The exclusion criteria were as follows: (1) no pathological confirmation of NPC; (2) poor patient compliance, incomplete data, or severe motion artifacts. Finally, 66 patients who were pathologically diagnosed with NPC were included in this study. Patients’ characteristics are presented in Table 1.

**Table 1:** Patients’ characteristics

Characteristics	Values
Number of patients (%)	66
Mean age ± SD (range); years	44 ± 11 (18–64)
Gender	
Male	44 (66.7%)
Female	22 (33.3%)
Histological type	
NKUC	66 (100%)
T stage	
T1	7 (10.6%)
T2	6 (9.1%)
T3	42 (63.6%)
T4	11 (16.7%)
N stage	
N0	1 (1.5%)
N1	30 (45.5%)
N2	18 (27.2%)
N3	17 (25.8%)
M stage	
M0	61 (92.4%)
M1	5 (7.6%)
AJCC stage	
I	2 (3%)
II	8 (12.1%)
III	30 (45.5%)
IV	26 (39.4%)

Abbreviations: *SD*, standard deviation; *NKUC*, nonkeratinizing undifferentiated carcinoma

### MRI acquisition

All MRI measurements were performed on a 3.0-T MR machine (uMR790; United Imaging Healthcare Co., Ltd) with a head and neck combined coil. Transverse T2-weighted FSE sequences and transversal, sagittal, and coronal plane T1-weighted FSE images were obtained before contrast injection. After the injection of gadopentetate dimeglumine at a dose of 0.1 mmol/kg, T1-weighted transverse, sagittal, and coronal sequences (with fat saturation) were obtained using parameters similar to pre-injection imaging.

Transverse T2-weighted FSE sequence, transverse T1-weighted FSE sequence, post-contrast transverse T1-weighted FSE sequence, and post-contrast coronal T1-weighted FSE with fat suppression were experimental sequences, which were obtained by both ACS and PI techniques, respectively. The periods of examinations were recorded for all patients. The parameters (TR, TE, ETL, etc.) used in the ACS sequences and PI sequences of the same patient are consistent; details of parameters of ACS and PI sequences are listed in Table 2.

**Table 2:** MRI sequences and parameters

Sequence parameter	Tra T2WI FSE ACS	Tra T2WI FSE PI	Tra T1WI FSE ACS	Tra T1WI FSE PI	Post-contrast Tra T1WI FSE ACS	Post-contrast Tra T1WI FSE PI	Post-contrast Cor T1WI FSE ACS	Post-contras Cor T1WI FSE PI
Fov (mm)	240 × 240	240 × 240	240 × 240	240 × 240	240 × 240	240 × 240	280 × 240	280 × 240
TR/TE (ms)	4800/120	4800/120	662/8.16	662/8.16	789/8.12	789/8.12	576/10.82	576/10.82
Matrix	384 × 269	384 × 269	384 × 307	384 × 307	384 × 307	384 × 307	352 × 226	352 × 226
ETL	28	28	2	2	2	2	4	4
Bandwidth (Hz)	260	260	280	280	250	250	250	250
Average	1	1	1	1	1	1	1.2	1.2
Number slices	40	40	40	40	40	40	24	24
Spatial resolution	0.89 × 0.63 × 5	0.89 × 0.63 × 5	0.78 × 0.63 × 5	0.78 × 0.63 × 5	0.78 × 0.63 × 5	0.78 × 0.63 × 5	1.06 × 0.8 × 2	1.06 × 0.8 × 2
Acquisition	ACS	PI	ACS	PI	ACS	PI	ACS	PI
Accelerating factors	2.25	2	2.25	2	2.25	2	2.5	2
Fat suppression	No	No	No	No	No	No	Yes	Yes

*ACS*, AI-assisted compressed sensing; *PI*, parallel imaging; *Tra T2WI*, transverse T2-weighted image; *Tra T1WI*, transverse T1-weighted image; *Cor T1WI*, coronal T1-weighted image

### ACS

**Fig. 1:**
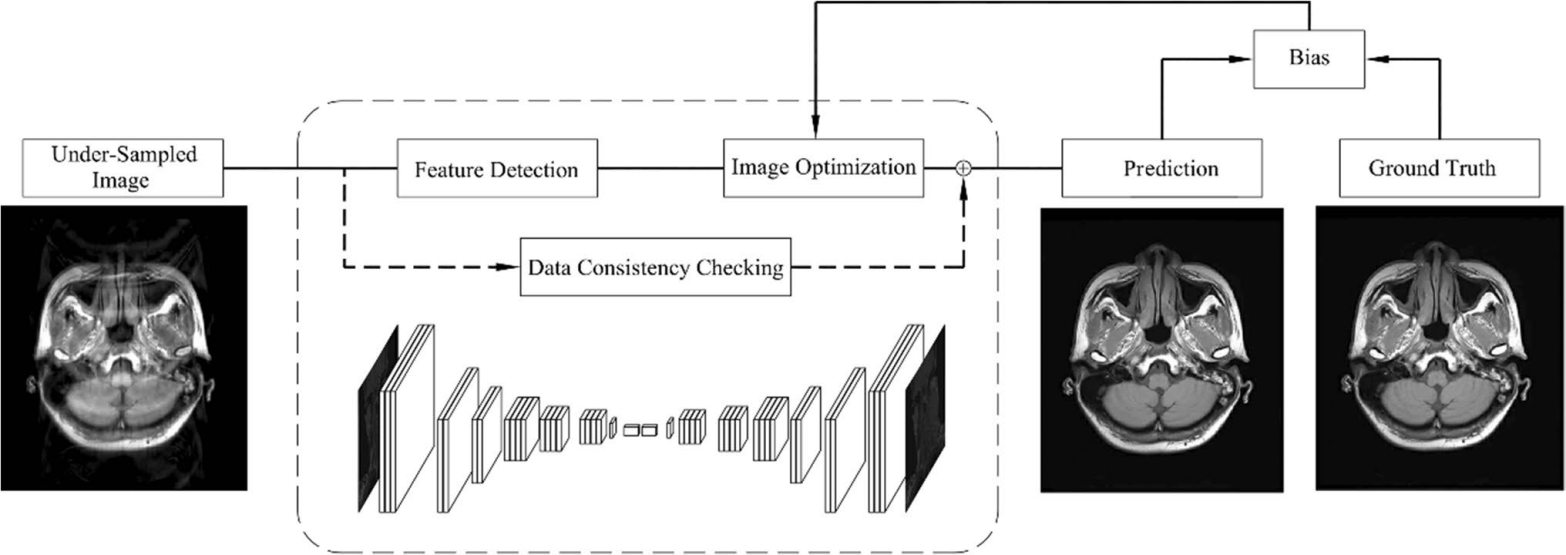
The AI module in ACS mitigates reconstruction artifacts at high acceleration levels

ACS incorporates CS, PI, HF, and AI to provide an improved MR acceleration solution. It innovatively introduces AI module based on deep neural networks. The goal of the AI module in ACS is to learn the features of fully sampled high-quality images without reconstruction artifacts, converting the obtained full k-space data into the image space, as the target output. The AI module is trained based on the designed Residual Neural Network (ResNet), which is widely used in the convolutional neural network (CNN) [17, 28–30]. The structure of the network consists of two convolutional operations and a skip connection. A long skip-over connection between the input and output of the network is added to learn the residual between the fully sampled and under-sampled images to improve the convergence rate during learning. To further improve the quality of the reconstructed images, a least-squares generative adversarial network training strategy is used [31]. The network design is shown in Fig. 1. The under-sampled images provide the information really obtained during the scanning process. The prior knowledge of the input data is maintained by the important Data Consistency Checking model in the internal network, while the parameters in the Feature Detection and Image Optimization processes will be optimized during the training phase until the prediction of the network reaches the optimal state. During the training phase of the AI model, a great number of full k-space data were collected and retrospectively under-sampled and converted to the image space as input. “This FDA-approved deep learning assisted reconstruction method was trained based on two million fully sampled slices previously acquired with phantom (2%) and volunteers (98%)” [31].

The compressed AI module is integrated into the iterative reconstruction program of the compressed sensing framework to get the final images. ACS follows the principle of compressed sensing and uses the learning ability of AI module at the same time. This effective combination fulfills the advantages of deep learning and also enables the AI module is controllable.

### Quantitative image analysis

The quantitative image analysis was performed on a workstation (uWS-MrR005; United Imaging Healthcare Co., Ltd.). The images generated by both ACS and PI techniques were required to measure the signal intensity (SI) and the standard deviation (SD) by placing in regions of interest (ROIs). ROIs were placed on two sets of images of transverse T2-weighted FSE sequence, transverse T1-weighted FSE sequence, post-contrast transverse T1-weighted FSE sequence, and post-contrast coronal T1-weighted FSE with fat suppression sequence. Then, the following data were measured at the largest lesion in both sets of images: The SI of the lesion, the SI of the lateral pterygoid muscle (ROI should be placed on the image of the contralateral lateral pterygoid muscle if the ipsilateral lateral pterygoid muscle has been invaded), the SD at the four corners (the top-left, top-right, bottom-left, and bottom-right corners) of background in the same layer image. The SNR and the CNR were calculated using the following formula [32]:

1 $$\mathrm{SNR}=\frac{0.66\times S}{SD}\times \sqrt{\frac{10}{d}}$$

where *S* is the SI lesion, *SD* is the mean standard deviation of the SI at the four corners of background noise, and *d* is the slice thickness.

To calculate the CNR, the following formula was applied:

2 $$\mathrm{CNR}=\frac{{SI}_{\mathrm{tissue}1}-{SI}_{\mathrm{tissue}2}}{SD}$$

where *SI*_tissue1_ and *SI*_tissue2_ are the SI of the lesion and the lateral pterygoid muscle, respectively; *SD* is the mean standard deviation of the signal intensity at the four corners of background noise.

MRI of the neck is particularly important in the evaluation of lymph node metastasis in NPC. The evaluation of the SI in the lower neck region was performed by measuring the SNR of the trapezius muscle. ROIs were placed on the two image sets (ACS and PI) of the trapezius muscle from the post-contrast coronal T1-weighted FSE with fat suppression sequence to obtain the SI of the trapezius muscle. When the SD at the four corner signals of background noise was measured, the mean and the SD were calculated. The SNR of the trapezius muscle was calculated using Eq. (1).

### Qualitative image analysis

The qualitative image analysis was carried out using the Picture Archiving and Communication System (PACS) (Centricity™ PACS; GE Medical Systems). All the imaging analyses were performed by two radiologists (H.L. and C.M.X.) who had 18 and 34 years of experience in the diagnosis of NPC. Two readers were individually blinded to patients’ clinical data and evaluated lesion detection, margin sharpness of lesions, artifacts, and overall image quality using a 5-point scoring system. In the present study, the lesion detection was scored as follows: 1, poor, almost invisible; 2, fair, lesions were partially visible; 3, moderate, lesions could be detected, while they were unclear; 4, good, lesions could be detected, and anatomical details were relatively clear; 5, excellent, lesions were easily detected, and anatomical details were very clear. The margin sharpness of lesions was rated as follows: 1, unreadable; 2, extremely blur; 3, moderately blur; 4, mildly blur; 5, no blur. The artifacts were scored as follows: 1, unreadable; 2, severe artifact; 3, moderate artifact; 4, mild artifact; 5, no artifact. The overall image quality was scored as follows: 1, poor; 2, fair; 3, moderate; 4, good; 5, excellent.

### Statistical analysis

The statistical analysis was carried out using SPSS 26.0 software (IBM). The examination time and the values of the SNR and CNR were compared between ACS and PI sequences. All measurement data were expressed as the mean ± SD. Normality of data was tested by the Kolmogorov-Smirnov test. The paired sample *t*-test was used for the analysis of normally distributed data, and the Wilcoxon signed-rank test was utilized for the analysis of abnormally distributed data. The image quality scores of sequences with ACS and PI techniques were also tested using the Wilcoxon signed-rank test. Weighted kappa statistic and *χ*2 test were used to evaluate the inter-observer agreement of imaging analyses for each qualitative indicator. The kappa coefficients for inter-observer agreements were interpreted as follows:  < 0.20, very weak; 0.21–0.40, weak; 0.41–0.60, moderate; 0.61–0.80, satisfactory; and 0.81–1.00, excellent. *p* < 0.05 was considered statistically significant.

## Results

**Fig. 2:**
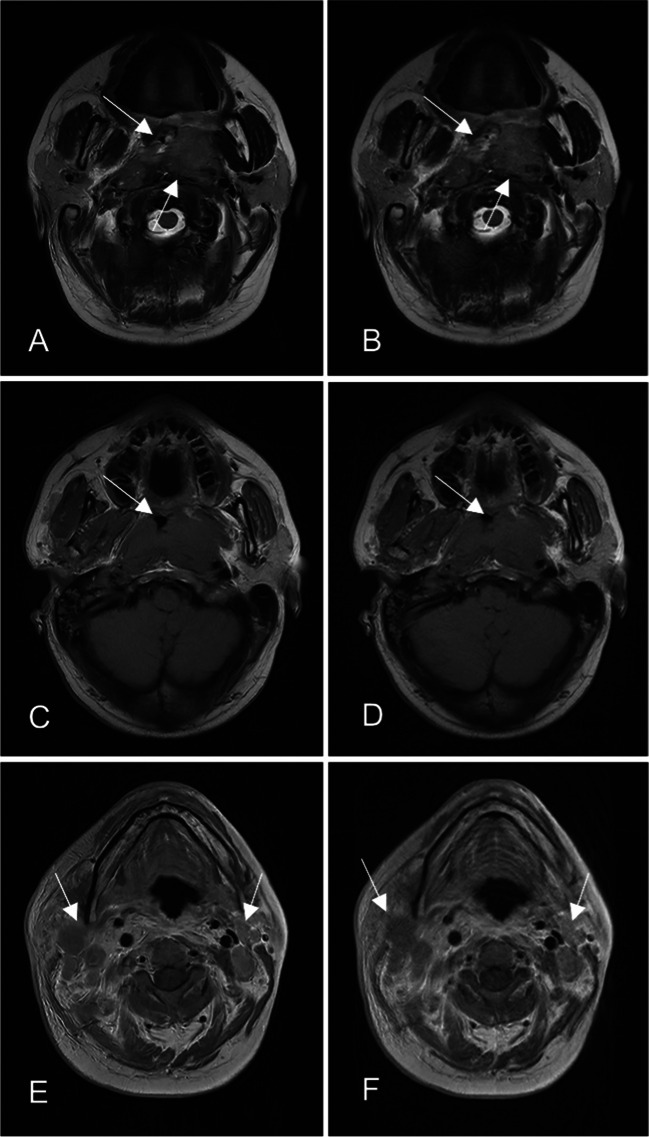
A 34-year-old male patient with NPC. The MRI sequences and examination time were summarized as follows: T2-weighted FSE sequences in the transverse planes: (**A**) with ACS technique (39.6 s); (**B**) with PI technique (59.0 s); T1-weighted FSE sequences in the transverse planes: (**C**) with ACS technique (74.8 s); (**D**) with PI technique (105.4 s); post-contrast T1-weighted FSE sequences in the transverse plane: (**E**) with ACS technique (92.1 s); (**F**) with PI technique (129.9 s). The border of NPC was clearer for FSE sequences with ACS (**A**, **C** arrows) than for FSE sequences with PI (**B**, **D** arrows); the scan time was shorter with ACS (**E**) than with PI (**F**), and the artifacts of swallowing motion could be effectively suppressed (**E**, **F** arrows) due to shortening of time

**Fig. 3:**
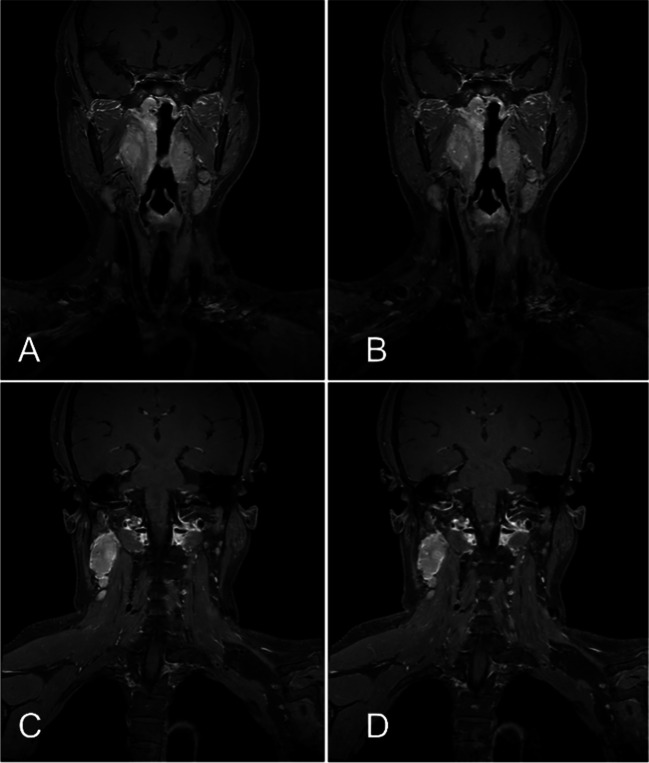
A 54-year-old female patient with NPC. The MRI sequences and examination time were summarized as follows: post-contrast T1-weighted FSE sequences in the coronal planes: (**A**) and (**C**) with ACS technique (124 s); (**B**) and (**D**) with PI technique (216 s); ACS provided a better image quality than the PI, while the scan time was reduced over 40%

Two representative cases are illustrated in Figs. 2 and 3.

**Fig. 4:**
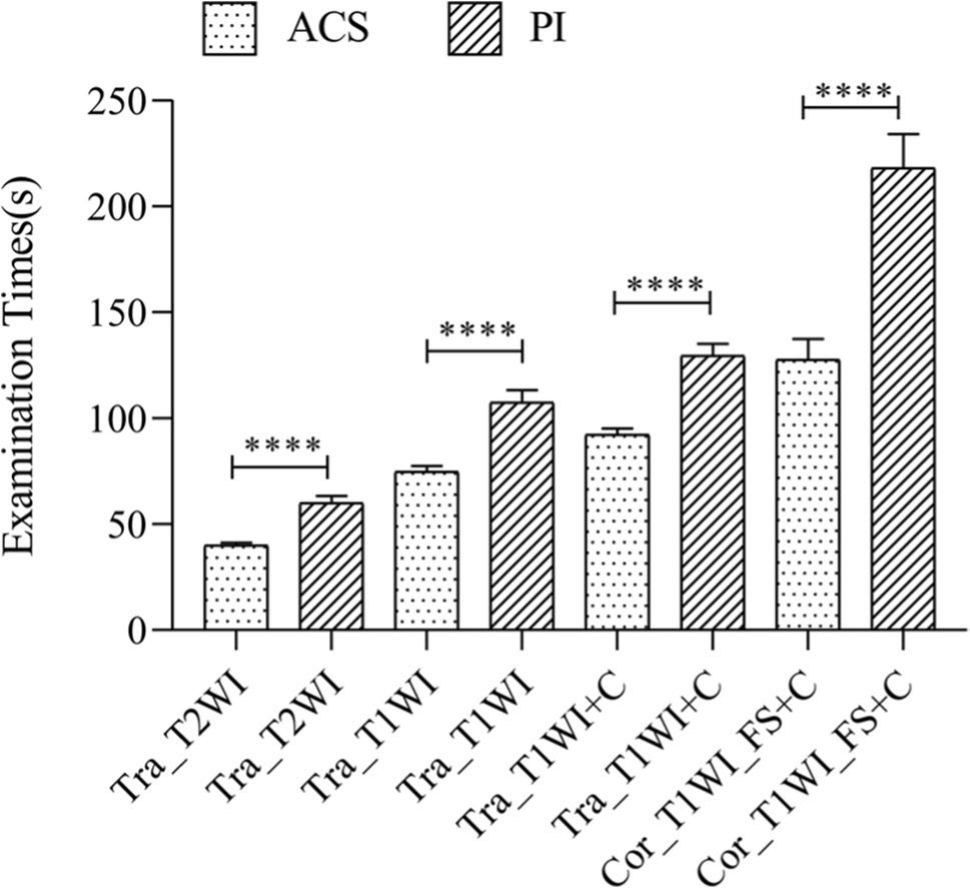
Comparison of examination time between ACS sequences and PI sequences

The comparison of examination time (Fig. 4) showed that the time of FSE sequences with ACS was significantly shorter than that of FSE sequences with PI, and the overall examination time of the ACS sequences and the PI sequences showed a statistical difference. The comparison of examination time showed that the time of T2-weighted sequences in the transverse planes with ACS was significantly shorter than that with PI (ACS (40.08 ± 1.33 s) vs. PI (60.21 ± 3.24 s), *p* < 0.0001). The time of T1-weighted sequences in the transverse planes with ACS was significantly shorter than that with PI (ACS (74.91 ± 2.76 s) vs. PI (107.54 ± 5.89 s), *p* < 0.0001). The time of post-contrast T1-weighted sequences in the transverse planes with ACS was significantly shorter than that with PI (ACS (92.40 ± 2.82 s) vs. PI (129.72 ± 5.66 s), *p* < 0.0001). The time of post-contrast T1-weighted sequences in the coronal planes with ACS was significantly shorter than that with PI (ACS (127.78 ± 9.74 s) vs. PI (218.21 ± 16.13 s), *p* < 0.0001). The total time of 4 sequences using ACS and PI was 335.17 ± 11.40 and 515.67 ± 21.80 s, respectively (*p* < 0.0001).

**Fig. 5:**
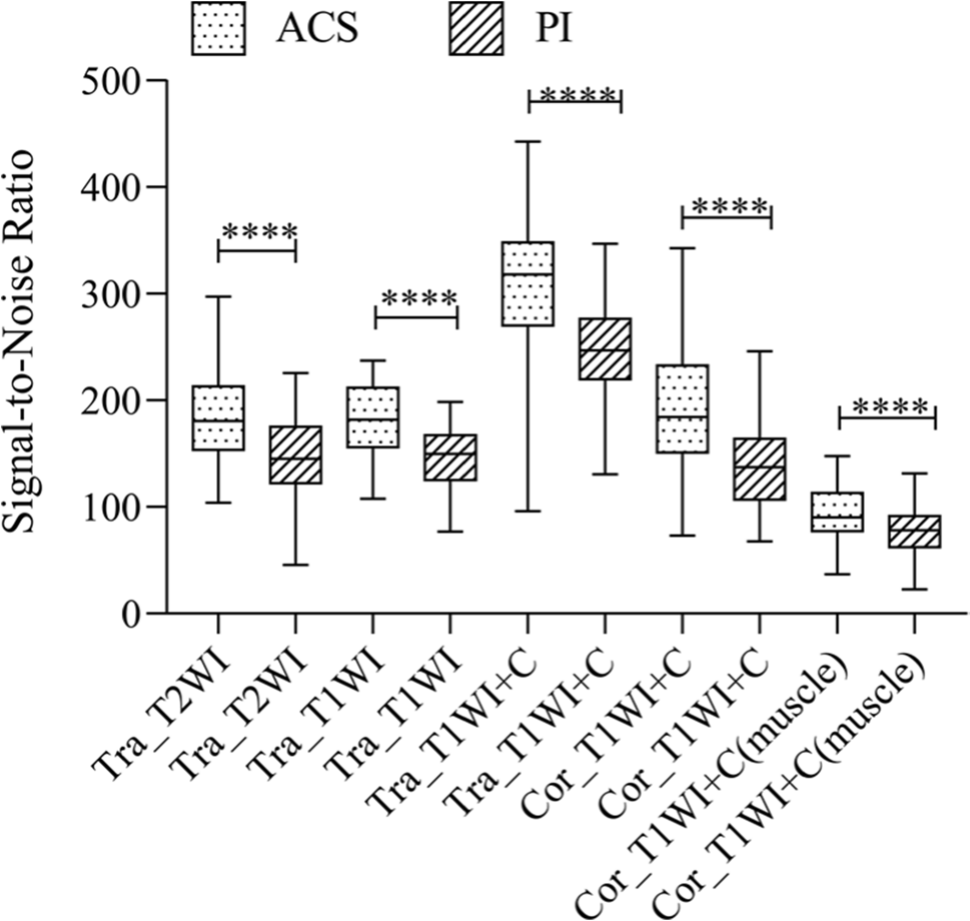
Comparison of SNR values between ACS sequences and PI sequences. ACS sequences were significantly higher than FSE sequences with PI technique

**Fig. 6:**
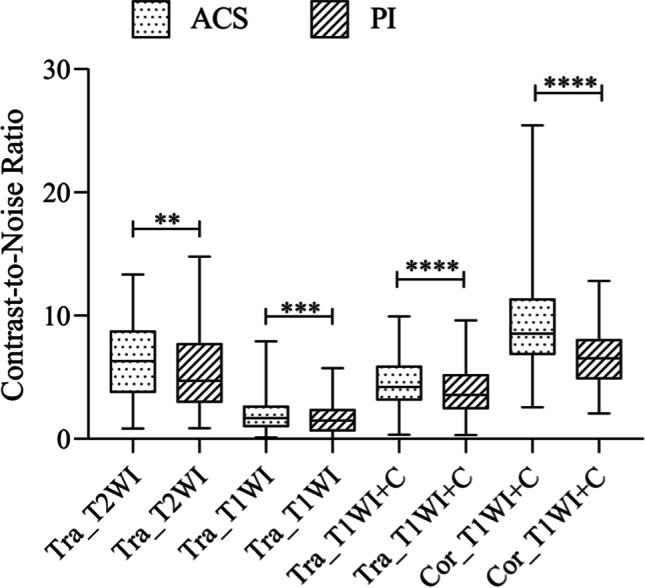
Comparison of CNR values between ACS sequences and PI sequences

The comparison of SNR (Table 3 and Fig. 5) showed that the SNR values of FSE sequences with ACS were significantly higher than those of FSE sequences with PI. The SNR values of T2-weighted sequences in the transverse planes with ACS were significantly higher than those with PI (ACS (182.07 ± 41.69) vs. PI (145.27 ± 38.05), *p* < 0.0001). The SNR values of T1-weighted sequences in the transverse planes with ACS were significantly higher than those with PI (ACS (183.53 ± 31.36) vs. PI (147.02 ± 27.11), *p* < 0.0001). The SNR values of post-contrast T1-weighted sequences in the transverse planes with ACS were significantly higher than those with PI (ACS (312.43 ± 56.59) vs. PI (245.89 ± 46.68), *p* < 0.0001). The SNR values of post-contrast T1-weighted sequences in the coronal planes with ACS were significantly higher than those with PI (ACS (189.05 ± 60.84) vs. PI (140.82 ± 43.26), *p* < 0.0001). The SNR values of the trapezius were also statistically significant in post-contrast coronal T1-weighted FSE with fat suppression sequences (ACS (94.76 ± 27.08) vs. PI (78.63 ± 23.00), *p* < 0.0001). As for CNR values, the FSE sequences with ACS were also significantly higher than those of FSE sequences with PI (Table 3 and Fig. 6). The CNR values of T2-weighted sequences in the transverse planes with ACS were significantly higher than those sequences with PI (ACS (6.21 ± 3.00) vs. PI (5.52 ± 3.05), *p* < 0.0027). The CNR values of T1-weighted sequences in the transverse planes with ACS were significantly higher than those sequences with PI (ACS (1.96 ± 1.38) vs. PI (1.59 ± 1.11), *p* < 0.0003). The CNR values of post-contrast T1-weighted sequences in the transverse planes with ACS were significantly higher than those sequences with PI (ACS (4.59 ± 2.19) vs. PI (3.93 ± 2.03), *p* < 0.0001). The CNR values of post-contrast T1-weighted sequences in the coronal planes with ACS were significantly higher than those sequences with PI (ACS (9.32 ± 4.07) vs. PI (6.70 ± 2.54), *p* < 0.0001).

**Table 3:** Quantitative comparison of SNR and CNR values between the two methods

	Method	ACS (mean ± SD)	PI (mean ± SD)	*p* value
SNR	Tra_T2WI_FSE	182.07 ± 41.69	145.27 ± 38.05	< 0.0001
	Tra_T1WI_FSE	183.53 ± 31.36	147.02 ± 27.11	< 0.0001
	Post contrast Tra_T1WI_FSE	312.43 ± 56.59	245.89 ± 46.68	< 0.0001
	Post contrast Cor_T1WI_FSE	189.05 ± 60.84	140.82 ± 43.26	< 0.0001
	Post contrast Cor_T1WI_FSE (muscle)	94.76 ± 27.08	78.63 ± 23.00	< 0.0001
CNR	Tra_T2WI_FSE	6.21 ± 3.00	5.52 ± 3.05	< 0.0027
	Tra_T1WI_FSE	1.96 ± 1.38	1.59 ± 1.11	< 0.0003
	Post contrast Tra_T1WI_FSE	4.59 ± 2.19	3.93 ± 2.03	< 0.0001
	Post contrast Cor_T1WI_FSE	9.32 ± 4.07	6.70 ± 2.54	< 0.0001

*SNR*, signal-to-noise ratio; *CNR*, contrast-to-noise ratio

*p* values were calculated using the paired sample *t*-test

Table 4 shows that the scores of lesion detection, margin sharpness of lesions, artifacts, and overall image quality are higher in the ACS sequences than those in the PI sequences (*p* (for all)  < 0.0001). The inter-observer agreement for the independent qualitative analysis showed satisfactory-to-excellent agreement, and *κ* value ranged from 0.627 to 0.892 (*p* (for all)  < 0.0001).

**Table 4:** Comparison of subjective evaluation scores and inter-observer kappa values between ACS sequences and PI sequences

Method	Lesion detection		Margin sharpness of lesions		Artifacts		Overall image quality	
	Subjective evaluation	Kappa	Subjective evaluation	Kappa	Subjective evaluation	Kappa	Subjective evaluation	Kappa
Tra_T2WI_ACS	4.89 ± 0.31	0.841	4.92 ± 0.27	0.784	4.88 ± 0.33	0.858	4.91 ± 0.29	0.817
Tra_T2WI_PI	3.86 ± 0.46	0.691	3.73 ± 0.45	0.773	4.11 ± 0.40	0.794	3.92 ± 0.38	0.780
Tra_T1WI_ACS	4.00 ± 0.21	0.660	4.08 ± 0.33	0.640	4.45 ± 0.56	0.892	4.02 ± 0.21	0.660
Tra_T1WI_PI	3.38 ± 0.49	0.627	3.11 ± 0.32	0.776	3.80 ± 0.42	0.719	3.36 ± 0.48	0.664
Tra_T1WI + C_ACS	4.85 ± 0.36	0.882	4.61 ± 0.53	0.851	3.95 ± 0.27	0.790	4.59 ± 0.52	0.822
Tra_T1WI + C_PI	3.93 ± 0.33	0.784	3.68 ± 0.48	0.867	3.30 ± 0.58	0.866	3.79 ± 0.43	0.831
Cor_T1WI + C_ACS	4.84 ± 0.41	0.727	4.84 ± 0.41	0.836	4.04 ± 0.26	0.884	4.83 ± 0.42	0.847
Cor_T1WI + C_PI	3.96 ± 0.31	0.756	3.84 ± 0.37	0.830	3.40 ± 0.58	0.868	3.88 ± 0.37	0.886

*p* values were calculated using the Wilcoxon signed rank test. All values were presented as mean ± standard deviation of the scores (*p* (for all) < 0.0001). Inter-observer agreement for image analyses was assessed by the weighted kappa values (*p* all < 0.0001)

## Discussion

The present study explored whether applying ACS would decrease the examination time and affect the MR image quality of NPC by comparing with the PI.

The quantitative assessment in the study showed that with applying both the ACS and conventional PI for two sets of FSE sequences on the same patient, the sequence scanning time using ACS was significantly shorter than that by PI at the same resolution. The time of 4 sequences with ACS was 180 s shorter than that with the conventional PI, indicating that time saving was approximately equal to 35%. Meanwhile, the SNR and CNR values of images did not descend drastically, and these values were even higher in the sequences that used ACS, which indicated that ACS has the ability to maintain image quality even at high acceleration factors. In previous combined nasopharyngeal neck scans, the SI of the lower neck was mainly poor due to the limitation of the head and neck coil and the vascular pulsation artifacts after contrast injection, especially the fat suppression sequences in the coronal plane. Hence, the SNR of the coronal fat suppression sequence was deliberately measured. It was found that the SNR of the trapezius muscle with ACS was higher than with the conventional PI, while the scan time was reduced over 40%, indicating that ACS could also maintain the imaging quality after the imaging velocity was raised. ACS can make a well-balanced relationship between the imaging velocity and imaging quality.

The qualitative assessment in the study showed that the scores of the sequences with ACS were higher than with PI in lesion detection, margin sharpness of lesions, artifacts, and overall image quality. Inter-observer agreement was evaluated for all qualitative indicators for each method, and the results showed that the values of kappa ranged from 0.627 to 0.892. Therefore, satisfactory-to-excellent agreement was achieved in the present study. We found that margin sharpness of NPC lesions was clearer for FSE sequences with ACS, and the ACS-based sequences could clearly distinguish muscles, mucosa membranes, and adipose tissues compared with the PI-based sequences. It could be advantageous to more clearly define the invasion range of the tumor tissues, especially for T2-weighted FSE sequences in the transverse planes. This characteristic may be related to the ability of ACS to suppress noises and reduce artifacts, resulting in a higher image clarity and a better overall quality than those achieved by the PI method. The PI might produce residual aliasing and noise-induced artifacts at high acceleration factors, which were previously reported [11, 33–36]. However, ACS has eliminated this challenge perfectly. The AI module in ACS was trained using a huge amount of fully sampled data to suppress various reconstruction artifacts introduced by conventional methods at high acceleration factors without affecting anatomical and pathological structures. Patients with NPC may easily produce swallowing motion artifacts in MR scans, and in severe cases, they may not be able to complete the entire examination. However, the motion artifacts caused by swallowing motions were significantly reduced in the sequences using ACS in the present study. The ultra-fast imaging of ACS, which is the inherent ability of freeze motions to effectively reduce the artifacts, could be an influential factor.

Unlike the PI method, there are potential risks existing when using ACS from the deep learning method, whose high precision heavily depends on the size and variety of the training data set. Although the current deep learning algorithms have already shown accurate reconstruction results, the results still lack stability. The issues of instability majorly cover the following aspects: (1) the instability of some small noise perturbations; (2) the instability of microstructure changes; (3) the instability of sample number difference. In medical imaging, stability and accurate image reconstruction methods are essential for disease diagnosis. Thus, it is very significant to ensure stable outputs while improving the accuracy of the algorithm. However, according to the current research, the capability of ACS in lesion detection is no less than that of PI. Taking the T2WI FSE sequence as the example, 0 cases were rated 1 and 2 (1, poor, almost invisible; 2, fair, lesions were partially visible) by both readers on the ACS and PI methods.

At present, deep learning–based MRI solutions are used to solve problems, such as artifact reduction, motion correction, and denoising, which are mainly divided into two categories: deep learning–based image reconstruction and deep learning–based image post-processing [37, 38]. The method of deep learning–based image post-processing uses deep learning reconstruction tools integrated into MRI, separating MRI signals from noises by deep learning algorithms, to enhance the signal intensity, while suppressing noises [18, 39]. The ACS is the method belonging to deep learning–based image reconstruction, integrating AI module into the iterative reconstruction process of the compression perception framework, and some researchers demonstrated that this deep learning method helps the reconstruction of CS and ensures high fidelity when acquisition duration prolongs [40–42].

There are still several shortcomings in the present study. Firstly, the small sample size might cause a selection bias in the measured values. Secondly, only NPC patients with definite pathological results and typical imaging manifestations were included, while patients with other nasopharyngeal lesions were excluded. Last but not least, although the ACS has the feature of noise reduction to decrease the artifacts, some inherent artifacts might still not be eliminated.

In conclusion, compared with the PI technique, the ACS technique for MR examination of NPC can not only reduce scanning time, but also improve image quality.

## Abbreviations

ACS: Artificial intelligence (AI)–assisted compressed sensing
AI: Artificial intelligence
AiCE: Advanced intelligent Clear IQ Engine
CNN: Convolutional neural network
CNR: Contrast-to-noise ratio
CS: Compression sensing
CT: Computed tomography
DLR: Deep learning reconstruction
FOV: Field-of-view
FSE: Fast spin-echo
HF: Half-Fourier
MRI: Magnetic resonance imaging
NKUC: Nonkeratinizing undifferentiated carcinoma
NPC: Nasopharyngeal carcinoma
PACS: Picture Archiving and Communication System
PI: Parallel imaging
ResNet: Residual Neural Network
ROI: Region of interest
SD: Standard deviation
SI: Signal intensity
SNR: Signal-to-noise ration

## Declarations

### Guarantor

The scientific guarantor of this publication is Guixiao Xu (corresponding author).

### Statistics and biometry

No complex statistical methods were necessary for this paper.

### Methodology

• retrospective

• observational study

• performed at one institution
